# *SMPD1* gene variants in patients with β-Thalassemia major

**DOI:** 10.1007/s11033-023-08275-x

**Published:** 2023-02-01

**Authors:** Fadime Ersoy Dursun, Filiz Özen

**Affiliations:** 1 Department of Hematology, Prof. Dr. Süleyman Yalçın City Hospital, Eğitim mah, Dr. Erkin Cd. No:161/1, 34722 Kadıköy, İstanbul, Turkey; 2 Department of Medical Genetics, Prof. Dr. Süleyman Yalçın City Hospital, Kadıköy, İstanbul, Turkey

**Keywords:** β-Thalassemia major, *SMPD1* gene variant, Acid sphingomyelinase, Chitotriosidase

## Abstract

**Background:**

β-thalassemia major and Niemann-Pick diseases have similar clinical and laboratory findings. We aimed to investigate the effects of sphingomyelin phosphodiesterase 1 (*SMPD1*) gene variants on the clinical and laboratory findings in patients with β-thalassemia major.

**Methods and results:**

This study included 45 patients who were followed up for β-thalassemia major in our clinic. Plasma chitotriosidase, leukocyte acid sphingomyelinase, liver enzymes, ferritin, hemogram, biochemical parameters, *SMPD1* gene variant analysis, cardiac T2* MRI, and liver R2 MRI were assessed in all patients. The *SMPD1* gene *c.132_143del, p.A46_L49del (c.108GCTGGC*[4] *(p.38AL*[4]*)) (rs3838786)* variant was detected in 9 of 45 (20.0%) patients. Plasma chitotriosidase, ferritin, acetyl aminotransferase, and alanine aminotransferase levels were significantly higher in patients with the gene variant than in those without (p < 0.05). Leukocyte acid sphingomyelinase levels were significantly lower in patients with the gene variant than in those without (p < 0.05).

**Conclusion:**

These results imply that the clinical and laboratory findings and some features of disease progression in patients with β-thalassemia major are similar to those of Niemann-Pick disease. They also suggest that *SMPD1* gene *c.132_143del, p.A46_L49del (c.108GCTGGC*[4] *(p.38AL*[4]*)) (rs3838786)* variant may underlie these clinical findings in patients with β-thalassemia major.

## Introduction

The frequency of β-thalassemia major (β-TM) carriers in Turkey is approximately 2.1% [1]. Similarly, Niemann-Pick disease (NPD) is a common glycogen storage disease in Turkey [2]. Both diseases are autosomal recessive conditions with similar clinical presentations, including anemia, splenomegaly, and skeletal involvement [3–6]. In addition to these clinical and laboratory similarities, the genes affected in both diseases are located on the 11th chromosome [7–9]. There could have been a misdiagnosis of NPD in some patients who were followed up because of a β-TM diagnosis [10].

Chitotriosidase (CTD; EC 3.2.1.14) is a functional chitinase that breaks down chitin, an important component of fungal and other pathogens [11, 12]. Chitotriosidase, synthesized by specifically activated macrophages and neutrophil precursors, is encoded by a gene on chromosome 1q31; it is regulated via an unknown molecular mechanism [11, 13–16]. Acid sphingomyelinase (ASM; EC 3.1.4.12) hydrolyzes the membrane lipid sphingomyelin to phosphorylcholine and bioactive lipid ceramide. Variants in the sphingomyelin phosphodiesterase 1 (*SMPD1*) gene lead to the type A and B forms of lysosomal storage disorder NPD [1, 17, 18].

The fact that the genetic loci of these two diseases are located on the same chromosome suggests that there may be a similarity and relationship between them [19, 20]. Thus, we hypothesized that there may be mutations in the *SMPD1* gene in patients with β-TM similar to that in patients with NPD. Variants in *SMPD1* gene may contribute to liver dysfunction and hepatosplenomegaly associated with multiple blood transfusions in patients with β-TM. There are some case reports in the literature on the coexistence of β-TM and NPD [9]. However, no study has shown the role of *SMPD1* variants in patients with β-TM to date. Therefore, a study of *SMPD1* variants in patients with β-TM and their impact on β-TM can be beneficial in understanding the pathology of β-TM. Interestingly, the *SMPD1* gene *c.132_143del, p.A46_L49del (c.108GCTGGC*[4] *(p.38AL*[4]*)) (rs3838786)* variant is considered potentially pathogenic in the literature [20]. This study was conducted to investigate the relationship between *SMPD1* gene *c.132_143del, p.A46_L49del (c.108GCTGGC*[4] *(p.38AL*[4]*)) (rs3838786)* variant and clinical and laboratory findings of the disease in patients with β-TM.

## Subjects and methods

### Patients

**Fig. 1:**
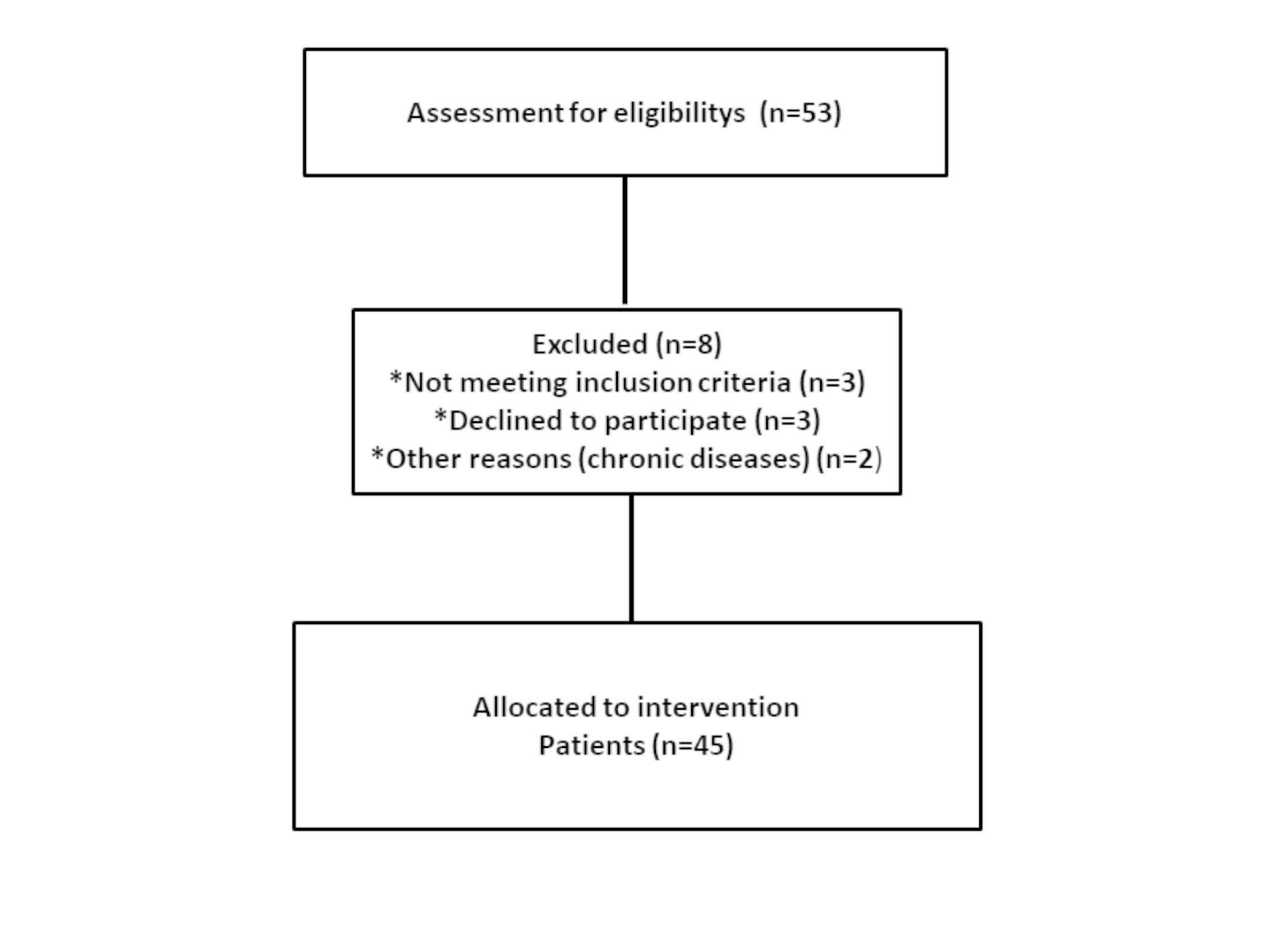
Flow diagram of the clinical trial of the patients included in the study

This prospective cohort study was performed in the hematology department of Prof. Dr. Suleyman Yalcin City Hospital in Istanbul, Turkey. The study included 45 patients (25 women and 20 men) diagnosed with β-TM between the ages of 18 and 53 years. A flow diagram of the clinical trial is shown in Fig. 1. The study was initiated with 53 patients with β-TM. During the study, eight patients were excluded from the study for the following reasons: three of them did not properly administer oral iron chelation therapy, three refused to continue the study, and two had other chronic diseases (diabetes mellitus and chronic renal failure). Thus, the study was continued with 45 patients. This study was approved by the local ethics committee of the Faculty of Medicine, Medeniyet University (No. 2021/0375). Written informed consent was obtained from each study participant in accordance with the principles of the World Medical Association Declaration of Helsinki after providing a data-oriented explanation regarding the aims and scope of the study.

All patients were confirmed to have β-TM based on their clinical findings and genetics. All patients typically received regular erythrocyte suspension transfusions every three weeks and continuous oral iron chelation therapy. The patients included in the study were selected among those who were regularly followed up by our department. Patients with other hematological or chronic diseases who did not agree to participate in the study, did not attend follow-up visits, or did not regularly receive chelation treatment were excluded.

Systemic examination findings, complete blood counts, biochemical analyses, ferritin levels, vitamin levels, thyroid function test results, and imaging test results collected during routine control patient examinations were retrospectively obtained from patient files and the hospital automation system at the Thalassemia Center of Prof. Dr. Süleyman Yalçın City Hospital.

### Enzyme assay

Plasma CTD activity and ASM levels were evaluated in patients with β-TM. For plasma CTD activity and ASM levels, 10 mL of blood was collected into EDTA tubes before blood transfusion.

The blood samples were rapidly transported to the laboratory. The plasma CTD activity was measured using a fluorometric method previously described [11, 13, 15]. In brief, 5 mL of plasma in an EDTA tube was incubated with 100 mL of 4-metilumbelliferyl chitotriosidase (Sigma Chemical Co., St. Louis, MO) at 37 °C in citrate/phosphate buffer (0.1/0.2 M; pH 5.2) for 15 min. The reaction was stopped by adding 2 mL of glycine/NaOH buffer (0.3 M; pH 10.6). The fluorescence of 4-methylumbelliferon was measured at 445 nm, and the results were expressed in µmol/L/h.

For the standard ASM assay, homogenates were prepared by sonication of the cell material in water [21–23]. Assays were performed in parallel, with and without unlabeled lysosphingomyelin. The reaction mixtures consisted of 10 µL homogenate with a standardized quantity of protein (10 µg for fibroblasts and 30 µg for leukocytes), 10 µL HMU-PC substrate, and either 10 µL LSM or 10 µL substrate buffer. The reaction mixtures were then incubated for 1 h at 37 °C (fibroblasts) or 17 h at 37 °C (leukocytes). Reactions were terminated by the addition of 200 µL stop buffer. For certain experiments, lysosphingomyelin was substituted with sphingomyelin (SM; Sigma, USA), and the effect of 0.2% Triton X-100 under these reaction conditions was studied. The fluorescence of 6-hexadecanoyl-4-methylumbelliferone was measured using a fluorimeter (Fluoro Count; Packard) with a filter set of 4-methylumbelliferone with excitation at 360 nm and emission at 460 nm. The fluorimeter was calibrated with 4-methylumbelliferone in the stop buffer.

### Gene variant analysis

Gene variant analyses in this study were performed at the Genetic Diseases Evaluation Center, Duzen Laboratory Group, Ankara, Turkey. Deoxyribonucleic acid (DNA) was isolated from patient samples to analyze *SMPD1* variants by Sanger sequencing and capillary electrophoresis following the relevant kit manual procedure with the MagNaPure LC 2.0 automatic isolation device (Roche diagnostic, California, USA). For DNA typing, Applied Biosystems 3500/3500xL Genetic Analyzer (Carlsbad, USA) was used [24]. Selective specific sequencing primers were designed to amplify all exons of the *SMPD1* gene from the isolated DNA and distinguish it from the pseudogene. Then, PCR, amplicon control, purification, sequencing PCR, loading of the samples into the device, and data analysis were performed. For the reference sequence NG_011780.1, using the NCBI/BLAST program, all coding sequences and intronic segments were created to be +/- 20.

### Statistical analyses

Statistical analysis was performed using SPSS software (Version 23.0, SPSS Inc., Chicago, IL, USA). If continuous variables were normal, they were described as the mean ± standard deviation (p > 0.05, Kolmogorov–Smirnov test or Shapiro–Wilk test (n < 30)), and if the continuous variables were not normal, they were described as the median. Continuous variables were compared using Student’s t-test or Mann–Whitney U test for parametric or nonparametric values, respectively. Categorical variables between the groups were analyzed using the chi-squared test or Fisher’s exact test. The level of statistical significance was set at p < 0.05.

## Results

The *SMPD1* gene was studied in 45 patients. Twenty-five female (55.6%) and 20 males (44.4%) patients, with a mean age of 26.8 ± 6.9 years (range: 18–53), were enrolled in the study. The laboratory findings, cardiac iron load, and hepatic iron load of the patients are presented in Table 1. The hemoglobin, platelet, LDH, total bilirubin, 25-(OH)-Vit D3, and ferritin levels were different from the reference values. Additionally, the mean plasma CTD and leukocyte ASM levels were evaluated and compared with normal reference values in some patients. The median plasma CTD activity was 91.1 (1.3–744.4) µmol/L/h and the leukocyte ASM level was 31.1 (2.3–91.8) nmol/mg/17 h. The mean plasma CTD activity was above the normal value (≥ 200 µmol/L/h) in 12 (26.7%) patients. Cardiac T2* magnetic resonance imaging (MRI) value was lower than 20 msn in 11 (24.4%) patients, and hepatic R2 value, obtained by MRI of the liver, was ≥ 7 mg/g in 13 (28.9%) patients. Furthermore, plasma ferritin levels in all patients were > 1000 ng/mL.

**Table 1:** Laboratory findings of the patients (n = 45)

Parameters	Results	Reference values
P-CTD (µmol/L/h)	91.1 (1.3-744.4)	< 200.0
L-ASM (nmol/mg/17 h)	31.1 (2.3–91.8)	> 10
Hemoglobin (gr/dL)	**8.5 ± 1.1**	**12–16**
Leukocytes (mm^3^)	13.3 ± 6.4	4.0–10.0
Platelet (mm^3^)	**659.1 ± 329.1**	**100–400**
MPV (fL)	9.8 ± 1.1	6–12
Glucose (mg/dL)	90.0 ± 15.1	70–105
Urea (mg/dL)	30.7 ± 7.1	19–44
Creatinine (mg/dL)	0.65 ± 0.1	0.72–1.25
Uric acid (mg/dL)	4.7 ± 1.4	3.5–7.2
Sodium (mmol/L)	137.6 ± 1.7	134–145
Potassium (mmol/L)	4.6 ± 0.4	3.5–5.1
Calcium (mg/dL)	9.4 ± 0.6	8.5–10.5
Magnesium (mg/dL)	2.0 ± 0.2	1.7–2.2
Phosphorus (mg/dL)	4.4 ± 0.9	2.3–4.7
AST (IU/L)	29.2 ± 17.1	< 37
ALT (IU/L)	30.3 ± 22.7	< 42
ALP (IU/L)	80.5 ± 40.3	< 260
GGT (IU/L)	18.1 ± 11.2	< 64
LDH (IU/L)	**233.9 ± 125.4**	**125–220**
Total Protein (gr/dL)	7.7 ± 0.6	6.4–8.3
Albumin (gr/dL)	4.6 ± 0.3	3.5–5.2
T. Bilirubin. (mg/dL)	**2.9 ± 1.4**	**0.2–1.2**
T. Cholesterol (mg/dL)	105.2 ± 30.5	< 200
Triglyceride (mg/dL)	130.0 ± 53.6	< 150
25 (OH) Vit D_3_ (ng/mL)	**17.5 ± 11.4**	**30–100**
Folic acid (ng/mL)	7.9 ± 7.9	3–20
Vitamin B12 (pg/mL)	355.8 ± 160.4	187–883
Ferritin (ng/mL)	**660.5 (47.1-18983.0)**	**4.4–207.0**
Cardiac T2* MRI (msn)	**31.8 (13.9–101.0)**	**> 20**
Liver R2 MRI (mg/g)	**44.8 (0.25–610.4)**	**3.2-7.0**

*Results are given as mean* ± *SD or median (min-max), SD: Standard deviation, P-CTD: Plasma Chitotriosidase, L-ASM: Leukocyte Acid Sphingomyelinase, MPV: Mean platelet volume, AST: Aspartate aminotransferase, ALT: Alanine aminotransferase, ALP: Alkaline phosphatase, GGT: Gamma-glutamyl transferase, LDH: Lactate dehydrogenase, MRI: Magnetic resonance imaging*

In this study, *SMPD1* gene *c.132_143del, p.A46_L49del (c.108GCTGGC*[4] *(p.38AL*[4]*)) (rs3838786)* variant was detected in nine (20.0%) patients. This gene has been recorded as pathological in Clin Var. In addition, some benign gene variants were detected (Table 2). No variants were detected in five patients. The patients were divided into two groups according to the *SMPD1* variant results: those with or without the variant (Table 3). The mean age of the patients with and without the variant was 26.9 ± 7.6 and 25.8 ± 4.6 years, respectively. There was no statistically significant difference in patient age between the two groups (p = 0.960). Plasma CTD, leukocyte ASM, ferritin, acetyl aminotransferase (AST), and alanine aminotransferase (ALT) levels were significantly different between patients with and without the *SMPD1* variant (p = 0.001, p = 0.004, p = 0.003, p = 0.009, and p = 0.017, respectively). In addition, the mean platelet volume was significantly higher in patients without the *SMPD1* variant (p = 0.035) than in those with the gene variant. No statistically significant differences were observed among the other parameters. Similarly, the liver R2 results and cardiac T2* values did not differ significantly between the two groups.

**Table 2:** SMPD1 gene variants in patients with -thalasemia major

β				
**Patients** **Number**	*SMPD1* Gene c.132_143del, p.A46_L49del (c.108GCTGGC[4] (p.38AL[4])) (rs3838786)	*SMPD1* Gene p.V36A (c.107T > C) (rs1050228) mutation	*SMPD1* Gene p.G508R (c.1522G > A) (rs1050239) mutation	*SMPD1* Gene c.138_143del, .A48_L49del (c.108GCTGGC[5] (p.38AL[5])) (rs3838786)
	**Pathogenicity**	**Benign allel**	**Benign allel**	**Benign allel**
1	**Homozygous**	Homozygous	-	-
2	**Homozygous**	Homozygous	-	-
3	**Homozygous**	Homozygous	-	-
4	**Homozygous**	Homozygous	-	-
5	**Heterozygous**	Homozygous	-	-
6	**Heterozygous**	Heterozygous	-	-
7	**Heterozygous**	Heterozygous	-	-
8	**Heterozygous**	-	-	-
9	**Heterozygous**	-	-	-
10	-	Homozygous	-	-
11	-	Homozygous	-	-
12	-	Homozygous	-	-
13	-	Homozygous	-	-
14	-	Homozygous	-	-
15	-	Homozygous	-	-
16	-	Homozygous	-	-
17	-	Homozygous	-	-
18	-	Homozygous	-	-
19	-	Homozygous	-	-
20	-	Homozygous	-	-
21	-	Homozygous	-	-
22	-	Homozygous	-	-
23	-	Homozygous	Homozygous	-
24	-	-	Homozygous	-
25	-	-	Homozygous	-
26	-	-	Homozygous	-
27	-	-	Homozygous	-
28	-	-	Homozygous	-
29	-	-	Homozygous	-
30	-	-	Homozygous	-
31	-	-	Heterozygous	Heterozygous
32	-	Heterozygous	Heterozygous	Heterozygous
33	-	Heterozygous	-	Heterozygous
34	-	Heterozygous	Heterozygous	-
35	-	Heterozygous	Heterozygous	-
36	-	Heterozygous	Heterozygous	-
37	-	Heterozygous	Heterozygous	-
38	-	Heterozygous		
39	-	Heterozygous		-
40	-	Heterozygous	-	-

*SMPD1: Sphingomyelin phosphodiesterase 1*

**Table 3:** Laboratory findings of the patients according pathological SMPD1 gene c.132_143del, p.A46_L49del (c.108GCTGGC[4] (p.38AL[4])) (rs3838786) variant

Parameters	*Pathological SMPD1* gene variant positive (n = 9)	*Pathological SMPD1* gene variant negative (n = 36)	p
Age	26.9 ± 7.6	25.8 ± 4.6	0.960
P-CTD (µmol/L/h)	320.8 (243-744.7)	91.9 (13.0-744.4)	**0.001**
L-ASM (nmol/mg/17 h)	11.4 (2.3–14.1)	33.6 (27.0-76.4)	**0.004**
Ferritin (ng/mL)	993.0 (467.5-18983)	689.19 (206.6–4320.0)	**0.003**
AST (IU/L)	36.5 (15–80)	22.0 (12–47)	**0.009**
ALT (IU/L)	38.0 (29–89)	15.5 (12–115)	**0.017**
ALP (IU/L)	111.0 (57-143.6)	78.2 (41–169.0)	0.151
Hemoglobin (gr/dL)	8.10 ± 1.05	8.62 ± 1.04	0.240
Leukocytes (mm^3^)	15.07 ± 6.4	12.86 ± 6.47	0.420
Platelet (mm^3^)	699.71 ± 339.98	649.67 ± 331.74	0.723
MPV (fL)	9.04 ± 0.66	9.99 ± 1.09	**0.035**
Uric acid (mg/dL)	4.53 ± 1.76	4.80 ± 1.35	0.653
Urea (mg/dL)	29.57 ± 7.96	30.97 ± 6.97	0.645
Creatinine (mg/dL)	0.63 ± 0.22	0.66 ± 0.13	0.670
Liver R2 MRI (mg/g)	8.00 (1.29–18.55)	3.95 (0.25-48.00)	0.784
Cardiac T2* MRI (msn)	24.50 (14.75–104.50)	26.8 (13.00-109.00)	0.471

*Results are given as mean* ± *SD or median (min-max), SMPD1: Sphingomyelin phosphodiesterase 1, P-CTD: Plasma Chitotriosidase, L-ASM: Leukocyte Acid Sphingomyelinase, AST: Aspartate aminotransferase, ALT: Alanine aminotransferase, ALP: Alkaline phosphatase, MPV: Mean platelet volume, MRI: Magnetic resonance imaging*

Liver enzymes, plasma CTD, leukocyte ASM, and ferritin levels of patients with the *SMPD1* variant are shown in Table 4. *SMPD* sequencing was performed on 45 patients with β-TM, and the *SMPD1* gene *c.132_143del, p.A46_L49del (c.108GCTGGC*[4] *(p.38AL*[4]*)) (rs3838786)* variant was detected in 9 of 45 (20%) patients. In the present study, four patients carrying this variant in *SMPD1* were homozygous, and five were heterozygous. AST and ALT levels were above the normal reference levels in all four homozygous patients. In contrast, ALT levels were elevated in only one of the five heterozygous patients with the gene variant, whereas AST and ALT levels were within the normal range in the others. Plasma CTD levels were above 200 µmol/L/h in all nine patients with the gene variant. Leukocyte ASM levels were below the reference values in only four patients with the gene variant.

**Table 4:** Some laboratory findings of patients with SMPD1 gene c.132_143del, p.A46_L49del (c.108GCTGGC[4] (p.38AL[4])) (rs3838786) variant

Patients	*SMPD1* Gene mutation	AST (IU/L)	ALT (IU/L)	ALP (IU/L)	P-CTD (µmol/L/h)	L-ASM (nmol/mg/17 h)	Ferritin (ng/mL)
Patient 1	Homozygous	**48.0**	**89.0**	100.0	**744.70**	3.1	18983.0
Patient 2	Homozygous	**80.0**	**76.0**	111.0	**564.90**	2.3	12223.2
Patient 3	Homozygous	**40.0**	**48.0**	65.0	**461.00**	2.3	5324.0
Patient 4	Homozygous	**45.0**	**38.0**	122.0	**320.80**	10.1	5688.6
Patient 5	Heterozygous	15.0	30.0	57.0	**246.60**	13.1	564.9
Patient 6	Heterozygous	32.0	**48.0**	111.0	**347.05**	13.0	993.0
Patient 7	Heterozygous	17.0	29.0	60.0	**303.80**	16.8	761.8
Patient 8	Heterozygous	26.2	32.9	132.3	**243.03**	11.1	548.8
Patient 9	Heterozygous	36.5	33.5	143.6	**263.60**	14.1	467.5

*SMPD1: Sphingomyelin phosphodiesterase 1, AST: Acetyl aminotransferase, ALT: Alanine aminotransferase, ALP: Alkaline phosphatase, P-CTD: Plasma Chitotriosidase, L-ASM: Leukocyte Acid Sphingomyelinase.*

The results of the correlation studies performed in patients with the *SMPD1* variant in this study are shown in Table 5. According to these results, a positive correlation was found between the plasma CTD and ferritin, AST, and ALT levels (r = 0.919, p = 0.001; r = 0.668 and p = 0.049; r = 0.968 and p = 0.001, respectively). In contrast, a negative correlation was found between leukocyte ASM levels and ferritin, AST, and ALT levels (r=-0.802, p = 0.009; r=-0.688, p = 0.048; and r=-0.779, p = 0.013, respectively).

**Table 5:** The relations of ferritin, P-CTD and L-ASM with other laboratory findings in patients with SMPD1 gene c.132_143del, p.A46_L49del (c.108GCTGGC[4] (p.38AL[4])) (rs3838786) variant

Parameters		P-CTD (µmol/L/h)	L-ASM (nmol/mg/17 h)
Ferritin (ng/mL)	r	0.919	-0.802
	p	**0.001**	**0.009**
AST (IU/L)	r	0.668	-0.688
	p	**0.049**	**0.048**
ALT (IU/L)	r	0.968	-0.779
	p	**0.001**	**0.013**
ALP (IU/L)	r	-0.072	0.163
	p	0.854	0.675
GGT (IU/L)	r	0.033	-0.027
	p	0.868	0.894
LDH (IU/L)	r	-0.138	0.107
	p	0.492	0.596

*P-CTD: Plasma Chitotriosidase, L-ASM: Leukocyte Acid Sphingomyelinase, AST: Aspartate aminotransferase, ALT: Alanine aminotransferase, ALP: Alkaline phosphatase, GGT: Gamma-glutamyl transferase, LDH: Lactate dehydrogenase*

## Discussion

We analyzed *SMPD1* gene variants in patients with β-TM. The *SMPD1* gene *c.132_143del, p.A46_L49del (c.108GCTGGC*[4] *(p.38AL*[4]*)) (rs3838786)* variant was detected in nine (20%) patients with β-TM. The results are novel because no such finding has been previously reported in the literature. Furthermore, we examined the effects of the *SMPD1* variant on clinical and laboratory findings in patients with β-TM. We detected high levels of liver enzymes (AST and ALT), ferritin, and plasma CTD and low leukocyte ASM levels in *SMPD1* variant-positive patients with β-TM.

A strong correlation exists between *SMPD1* gene variants and L-ASM levels, and these gene variants are detected in patients with NPD [25, 26], as in the case of an 11-year-old patient with NPD with a rare mutation in *SMPD1* [27]. However, no such studies have been conducted in patients with β-TM. In our study, L-ASM levels were lower in patients with the *SMPD1* variant than in those without the gene variant. These results show that the *SMPD1* variant may be important for disease progression in patients with β-TM. This study is the first to report the relationship between *SMPD1* gene variants and β-TM, which should be considered in patients with β-TM.

Liver enzyme levels increase in patients with β-TM [28]. Patients with β-TM develop cirrhosis over time owing to iron accumulation [29, 30]. The role of the *SMPD1* gene *c.132_143del, p.A46_L49del (c.108GCTGGC*[4] *(p.38AL*[4]*)) (rs3838786)* variant in the development of cirrhosis remains to be studied. Recently, liver disease has been reported to be an important cause of death in patients with β-TM [31]. However, the cause of liver disease in patients with β-TM has not been fully elucidated. Life-long transfusion regimens are required to alleviate anemia in patients with β-TM. Although regular blood transfusions have greatly improved the prognosis, iron overload, especially in the liver, poses a real threat to the quality of life of patients with β-TM [32]. A genetic feature other than blood transfusion may contribute to this condition. Hepatic siderosis does not develop in all patients with β-TM despite intensive blood transfusions. In this study, liver enzyme levels were higher in patients with the *SMPD1* variant than in those without. There were statistically significant differences in AST and ALT levels between patients with the *SMPD1* variant and those without this gene variant. The differences in actual values between the two groups were not very large and were not considered clinically significant. The patients included in our study were selected among those who were regularly followed up by our department. Notably, the levels of liver enzymes were higher in patients with the *SMPD1* variant than in those without the gene variant. This indicated that this gene variant is important for the progression of liver disease.

Variants in the *SMPD1* gene are associated with liver function in patients with NPD [33–35]. Homozygous and compound heterozygous patients with NPD have a more severe course than heterozygous patients [36–39]. In our study, liver enzyme levels were significantly higher in four homozygous patients than in heterozygous patients. In addition, L-ASM levels were significantly lower in homozygous patients than in heterozygous patients. These results indicate that homo- or heterozygosity of the gene mutation affects the degree of disease progression.

A relationship exists between *SMPD1* variants and L-ASM levels in NPD [40, 41]. However, such an association has not been reported in patients with β-TM. In the correlation tests performed in nine patients with the *SMPD1* variant, plasma CTD positively correlated with ferritin, AST, and ALT, implying that as plasma CTD and ferritin levels increased in these patients, AST and ALT levels also increased. In contrast, a negative correlation was found between leukocyte ASM and ferritin, AST, and ALT. These results suggest that the *SMPD1* variant causes an increase in plasma CTD, ferritin, AST, and ALT levels and a decrease in leukocyte ASM levels, similar to that in NPD.

Variant *c.132_143del, p.A46_L49del (c.108GCTGGC*[4] *(p.38AL*[4]*)) (rs3838786)* can cause various changes in the *SMPD1* gene. With this variant, valine is transformed into alanine. As a result, the protein structure is impaired; however, this is a benign change that does not impair protein synthesis. In addition, there were 12 base deletions from 32 to 143 bp in this gene. This change caused a deletion of four codons from the 46th position of the protein. This event was registered as a pathogen in ClinVar, albeit with mixed effects; 20 out of 50 cases were sick, and 30 were healthy, according to xxx. Although the effect the gene variant detected in our study has on β-TM can be open to a contradictory interpretation, we considered it to be pathological due to its clinical effect. In our opinion, the deletion mutation has a pathological effect in the clinic by disrupting protein synthesis. Similarly, in our study, we found a variant with a pathogenic effect in the clinic. This mutation should be studied further by accumulating data from more patients to determine whether it is a pathogenic gene variant.

This study had some limitations. First, a control group was not created due to financial constraints. However, we used normal reference values for leukocyte ASM and plasma CTD levels as alternatives. The second limitation was the limited number of patients.

## Conclusion

In conclusion, the most important result of this study was the detection of *SMPD1* gene *c.132_143del, p.A46_L49del (c.108GCTGGC*[4] *(p.38AL*[4]*)) (rs3838786)* variant in nine (20%) patients with β-TM. Several factors play a role in liver cirrhosis in patients with β-TM. Mutations in *SMPD1* in these patients may be related to increased levels of liver enzymes. Particularly, homozygous mutations may trigger this event. In addition, our findings suggest that some patients who were followed up with a diagnosis of β-TM may have a lysosomal storage disease, such as NPD. Therefore, it is necessary to refer patients with β-TM to metabolic specialists during the follow-up. We referred our patients to a metabolic specialist to monitor the possible effects of the *SMPD1* variant we detected. This study is the first to examine *SMPD1* variants in patients with β-TM. Importantly, this study also showed that liver enzyme and ferritin levels are high in patients with the *SMPD1* variant. These results support the need for further investigation of this subject with larger series of multicenter studies.

## Data Availability

The data supporting the findings of this study are available from the corresponding author upon reasonable request.

## Abbreviations

β-TM: β-Thalassemia major
NPD: Niemann-Pick disease
CTD: Chitotriosidase
ASM: Acid sphingomyelinase
SMPD1: Sphingomyelin phosphodiesterase 1
